# Insights into the resistance of a synthetically-derived wheat to Septoria tritici blotch disease: less is more

**DOI:** 10.1186/s12870-020-02612-z

**Published:** 2020-09-03

**Authors:** Harriet R. Benbow, Ciarán J. Brennan, Binbin Zhou, Thalia Christodoulou, Simon Berry, Cristobal Uauy, Ewen Mullins, Fiona M. Doohan

**Affiliations:** 1 UCD School of Biology and Environmental Science, University College Dublin, UCD Belfield, Dublin 4, Ireland; 2 UCD Earth Institute, University College Dublin, UCD Belfield, Dublin 4, Ireland; 3 UCD Centre for Plant Science, University College Dublin, UCD Belfield, Dublin 4, Ireland; 4 Limagrain UK Ltd, Windmill Avenue, Woolpit, Suffolk, IP30 9UP UK; 5 John Innes Centre, Norwich Research Park, Norwich, UK; 6 Teagasc Crops Research, Oak Park, Co. Carlow, Ireland

**Keywords:** wheat_1_, septoria_2_, transcriptomics_3_, Latent phase_4_, *Zymoseptoria tritici*_*5*_

## Abstract

**Background:**

Little is known about the initial, symptomless (latent) phase of the devastating wheat disease Septoria tritici blotch. However, speculations as to its impact on fungal success and disease severity in the field have suggested that a long latent phase is beneficial to the host and can reduce inoculum build up in the field over a growing season. The winter wheat cultivar Stigg is derived from a synthetic hexaploid wheat and contains introgressions from wild tetraploid wheat *Triticum turgidum subsp. dicoccoides*, which contribute to cv. Stigg’s exceptional STB resistance, hallmarked by a long latent phase. We compared the early transcriptomic response to *Zymoseptoria tritici* of cv. Stigg to a susceptible wheat cultivar, to elucidate the mechanisms of and differences in pathogen recognition and disease response in these two hosts.

**Results:**

The STB-susceptible cultivar Longbow responds to *Z. tritici* infection with a stress response, including activation of hormone-responsive transcription factors, post translational modifications, and response to oxidative stress. The activation of key genes associated with these pathways in cv. Longbow was independently observed in a second susceptible wheat cultivar based on an independent gene expression study. By comparison, cv. Stigg is apathetic in response to STB, and appears to fail to activate a range of defence pathways that cv. Longbow employs. Stigg also displays some evidence of sub-genome bias in its response to *Z. tritici* infection, whereas the susceptible cv. Longbow shows even distribution of *Z. tritici* responsive genes across the three wheat sub-genomes.

**Conclusions:**

We identify a suite of disease response genes that are involved in early pathogen response in susceptible wheat cultivars that may ultimately lead to susceptibility. In comparison, we hypothesise that rather than an active defence response to stave off disease progression, cv. Stigg’s defence strategy is molecular lethargy, or a lower-amplitude of pathogen recognition that may stem from cv. Stigg’s wild wheat-derived ancestry. Overall, we present insights into cv. Stigg’s exceptional resistance to STB, and present key biological processes for further characterisation in this pathosystem.

## Background

Wheat, *Triticum aestivum*, is one of the most important crops in the world and is the dominant crop in Europe. More land is dedicated to wheat production in the European Union than any other plant species, with 150 million tonnes of wheat grown across 26 million hectares of the European Union in 2017 (FAOSTAT, 2019). Wheat production is, however, challenged by a range of stresses, including fungal pathogens that can severely reduce both the yield and quality of wheat crops [1]. In Europe, one of the major antagonists of bread wheat production is Septoria Tritici Blotch (STB) [2], a foliar disease caused by the haploid, pathogenic fungus *Zymoseptoria tritici* (formerly known as *Mycosphaerella graminicola;* anamorph: *Septoria tritici*). STB symptoms manifest on the leaves as chlorotic and necrotic blotches, which reduce the photosynthetic capacity of the plant, leading to yield losses of up to 20% in the absence of adequate control [3].

At present, growers are reliant on chemical methods to control for STB (available in the form of four main fungicides: quinone-outside inhibitors (QoIs), sterol 14α-demethylation inhibitors (DMIs), succinate dehydrogenase inhibitors (SDHIs), and multi-site inhibitors [4]). Indeed, the magnitude of the STB problem in Europe is evident by the fact that up to 70% of fungicide usage is aimed at controlling STB [5]. However, this high dependency on fungicides has served as a strong driver of selection within European *Z. tritici* populations, resulting in the widespread emergence of fungicide-resistance, thus reducing the efficacy of fungicides in the field [6, 7]. Similarly, the introduction of STB-resistant varieties into agricultural systems has driven the evolution of *Z. tritici* to overcome host resistance [8]. Therefore, strategies for controlling STB disease are now multifaceted, combining resistance gene/genetic loci discovery and breeding for resistance [9–11], integrated pest management systems [12], innovations in fungicide chemistry [4], and the exploration of biological control [13].

Paramount to identifying novel sources of genetic resistance is the need to fully understand the life cycle of the pathogen and its interaction with the host. Approximately 3 h after contact with the leaf surface, *Z. tritici* spores germinate and the fungus penetrates the leaf through the stomata anywhere between 12 h and 10 days post infection (DPI) [14, 15]. The fungus grows in the sub-stomatal cavity and spreads through the apoplast to neighbouring substomatal spaces [16], before host cells begin to die and the fungus starts to feed necrotrophically [14, 17]. It is the function and impact of this latent phase, which precedes the switch to necrotrophy that remains elusive.

In fact, it has been suggested that the latent phase may be an artefact of evolution, as *Z. tritici* appears to have more genetic similarity with endophytes than other pathogens [18]. There is little evidence of nutrient acquisition from the host during the latent phase [14, 19], confuting suggestions that the fungus is feeding biotrophically. However, the latent phase does appear to impact the asexual fecundity of the pathogen; shortening the latent phase by silencing the plant homeodomain protein TaR1 allows the disease to progress to necrotrophy earlier, but reduces asexual sporulation of the fungus [20], whereas absence of some of the *Z. tritici* accessory chromosomes also brings forward the switch to necrotrophy but leads to an increase in numbers of pycnidia [21]. Conversely, the long latent phase observed in STB-resistant varieties does not appear to increase the ultimate levels of pycnidiospores generated beyond that of susceptible varieties; but it increases the time taken until pycnidia formation, which reduces inoculum build up over a growing season [22]. Given that the latent phase affects asexual spore production and abundance, and that up to 70% of the *Z. tritici* population in the field at the end of a growing season results from asexual reproduction [3], the interaction between host and pathogen during the latent phase is undoubtedly an important consideration for elucidating STB resistance mechanisms. However, a multitude of factors influence the duration of the latent phase, including host genotype, varietal growth stage when infected, environmental conditions, inoculum isolate and inoculum density [14, 22].

One genotype that displays highly effective field resistance in low, medium and high disease pressure environments is the synthetically-derived (derived from artificially created hexaploids) cultivar (cv.) Stigg [22]. The length of the latent phase in cv. Stigg under high-pressure field environments averages 36 days and leads to low STB disease progression. By contrast, the cv. Longbow is susceptible to STB infection [23], and has a much shorter latent phase (~ 12 days). Using these two cultivars, the primary goal of this study was to examine the molecular mechanisms involved in resistance versus susceptibility in the early stages of STB infection of hexaploid winter wheat, and to elucidate the transcriptomic response of cv. Stigg during infection that may contribute to its’ exceptional resistance to STB. Based on RNAseq of cvs. Stigg and Longbow during the early stages of STB infection (6–96 h post-inoculation with *Z. tritici*), we identify differences in the response to STB of these two cultivars and identify biological processes that are potentially involved in resistance and susceptibility to STB.

## Results

### Disease assessment of Stigg and longbow in response to *Z. tritici*

**Fig. 1:**
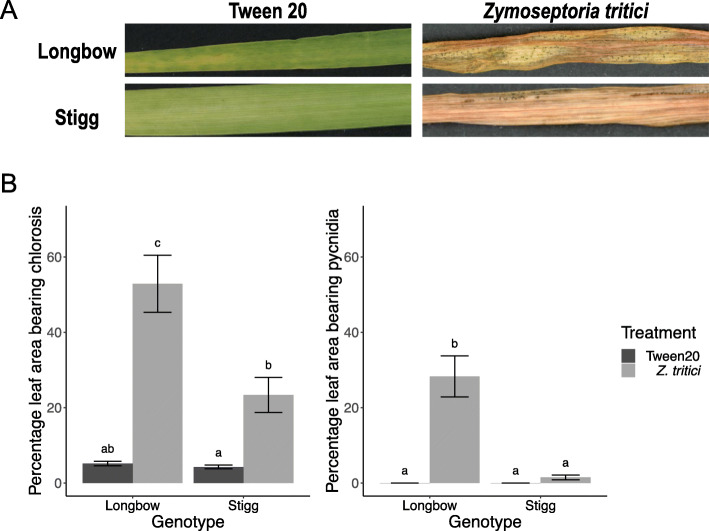
Assessment of Septoria tritici blotch (STB) disease levels on wheat cvs. Stigg and Longbow and Stigg. The 3rd leaf was treated with a suspension of *Zymoseptoria tritici* spores (or mock Tween20 solution) and after 28 days, **a** the disease was visualized and **b** the percentage of the leaf area bearing chlorosis pycnidia was determined. Both cvs. Longbow and Stigg show necrosis of the leaf, but development of pycnidia was significantly higher on Longbow leaves compared to Stigg leaves. Letters above bars indicate homogeneous subsets (bars that do not share a letter are significantly different from each other). Bars indicate SEM, *n* = 18 (6 per trial)

Disease assessments conducted on plants grown alongside those used for RNA sequencing validated that disease developed as expected within the trials conducted and that cv. Stigg was more resistant to STB than cv. Longbow. Furthermore, we observed that cv. Stigg’s resistance held up against an aggressive Irish isolate of *Z. tritici*, ‘Cork Cordiale 4″. At 28 DPI, an average of 52.8% of the leaf area in cv. Longbow was chlorotic, significantly higher (*P* < 0.05) than the chlorotic leaf area of cv. Stigg, 23.3%. 28% of the leaf surface area of cv. Longbow plants was covered in pycnidia, significantly higher (*P* < 0.05) than in cv. Stigg, where 1.5% of the cv. Stigg leaves bore pycnidia at this time. No pycnidia appeared on the control plants (treated with Tween20), and in both cultivars the chlorotic leaf area and leaf area bearing pycnidia was significantly higher in the treated plants versus the control plants (Fig. 1). No symptoms were observed during the first 96 h (the period during which tissue was collected for RNA sequencing).

### RNA sequencing and differential expression

**Fig. 2:**
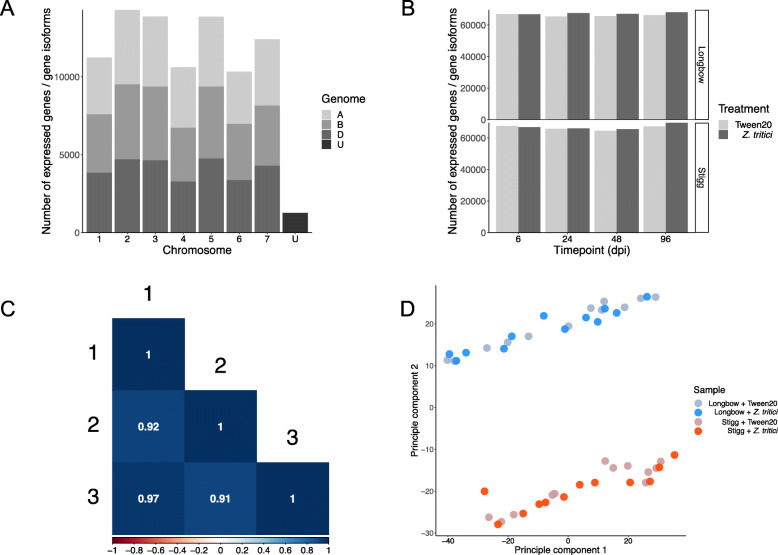
**a** Chromosome designation of all expressed *Triticum aestivum* genes across 48 RNAseq samples. Genes were evenly distributed across the A, B and D genome (~ 32% of all genes came from each genome). Chromosome groups 2, 3 and 5 provided the highest numbers of expressed genes, mirroring the chromosomal distribution of all genes across the wheat genome. **b** The number of expressed genes across each cultivar x timepoint x treatment combination. **c** correlation analysis of gene expressed between the three independent trials of the RNAseq experiment. The Pearson’s correlation coefficient is written within each box to represent each pairwise correlation. **d** A principle component analysis of the gene expression data across all 48 RNAseq samples. Clusters represent Genotype

A total of 5.9 billion 100 base pair reads were generated over 96 files (2 x genotypes, 2 x treatments, 4 x timepoints, 3 x trials x paired end reads), with an average of 60.9 million reads per sample. All samples had a mean phred score of 30 or greater at every base in each read. Reads were aligned to a reference index containing the gene annotation of wheat (IWGSC v1.1 *T. aestivum* high-confidence gene annotation, with every transcript (gene variant) of each gene represented) and *Z. tritici* (*Z. tritici* MG2 reference cDNA annotation) and read abundance was calculated using Kallisto. The mapped RNAseq data is represented as the number of genes expressed, and the number of unique gene variants expressed per gene. For each sample, a gene was considered expressed if it had a transcripts per million (TPM) value > 0.5 [24] in 2 out of the three trials. Across all samples, 87,888 genes/gene isoforms (71,636 unique wheat genes) were expressed, with an average of 2.45 variants expressed per gene. Expressed *T. aestivum* transcripts were evenly distributed across the three wheat genomes (A, B and D), with 32.8% from the A genome, 32.8% from the B genome, and 32.9% from the B genome. The remaining 1.4% of transcripts were unassigned to a chromosome. Similarly, the transcripts were evenly distributed across the 7 chromosome groups (1–7). Groups 2, 3 and 5 had the highest number of expressed genes (Fig. 2a), mirroring the chromosomal distribution of genes in the whole wheat genome, (where the group 2, 3 and 5 chromosomes contain the most genes). The number of expressed genes was even across all of the samples (Fig. 2b), with an average of 66,699 genes/gene isoforms expressed per sample (genotype x treatment x timepoint combination). A Pearson’s correlation analysis was used to test the correlation of gene expression between the three trials. Correlation was strong (correlation coefficient > 0.9, *P* < 0.05) between all three trials (Fig. 2c). A principle component analysis showed a distinct split of the samples into two main groups. These groups represent genotype, and there appears to be no clear division of the samples based on treatment, timepoint or trial (Fig. 2d).

From the pathogen perspective a total of 8961 *Z. tritici* genes were expressed across all samples, with an average of 2554 genes expressed. The number of expressed *Z. tritici* genes in the treated samples varied across trials (Additional file 1), and at the sequence depth used, the abundance of pathogen transcripts was 1.7% of the total reads (i.e. mapped to the *Z. tritici* reference), and the average transcripts per million value for the *Z. tritici* genes was 6.4 TPM (mean and median)). In comparison, the average TPM for wheat reads was higher, with a mean of 657 TPM and median of 34 TPM. Across the mock-treated samples, a low background average of 39 conserved fungal/potentially *Z. tritici* genes were expressed, indicating either a low level of background contamination, or misalignment of genes that are conserved between wheat and *Z. tritici* and therefore can map to the wrong reference.

A total of 586 high-confidence wheat genes/gene isoforms, representing 575 wheat genes were significantly differentially expressed (DEGs) (− 1 > = Log_2_ fold change > = 1; FDR-adjusted *P*-value < 0.05) between wheat samples treated with *Z. tritici* versus the mock solution of Tween20, in at least one comparison between treatments within each cultivar and each timepoint (Additional file 2). Of the differentially expressed transcripts, 230 were differentially expressed only in cv. Longbow, and 196 were differentially expressed only in cv. Stigg. The remaining 160 were differentially expressed in both cultivars at one or more of the four timepoints (Table 1).

**Table 1:** The number of differentially expressed transcripts by treatment and genotype in response to *Z. tritici* in wheat across the wheat sub-genomes

		Longbow					Stigg					Common				
Regulation	Timepoint	A	B	D	U	Total	A	B	D	U	Total	A	B	D	U	Total
Down	6	15	7	8	3	33	18	10	13	1	42	0	0	1	0	1
	24	16	10	14	0	40	29	14	19	0	62	0	1	5	1	7
	48	10	12	12	0	34	20	14	16	1	51	2	0	0	0	2
	96	15	12	18	1	46	11	5	14	1	31	1	1	0	0	2
Up	6	26	22	27	1	76	8	15	19	1	43	1	2	1	0	4
	24	41	26	37	1	105	18	9	12	0	39	3	4	4	1	12
	48	31	11	15	1	58	25	34	35	2	96	5	2	6	0	13
	96	25	18	22	2	67	27	8	20	1	56	1	0	4	0	5

The differentially expressed genes (by treatment) were distributed across all three wheat sub-genomes (A, B and D). A-genome derived genes made up 37% of the DEGs, compared to 25.6% B-genome and 35.2% D-genome derived. This sub-genome ratio of DEGs across each genome was significantly different from the expected percentage breakdown of 32:32:32 (χ^2^ *P*-value = 0.01). When the genome-distribution of DEGs was examined between the two cultivars, it was observed that the distribution in cv. Longbow is not different from the expected ratio, but it was in cv. Stigg (χ^2^ *P*-value = 0.02). Although the overall distribution of DEGs favoured the A-genome, in cv. Stigg, we observed a decrease in up-regulated A-genome derived DEGs, and a higher than expected number of DEGs from the B and D genomes, especially at 6 and 48 h post-inoculation.

A table of the top 5 differentially expressed genes in each condition can be seen in Table 2.

**Table 2:** The top 5 differentially expressed genes in each cultivar/timepoint

Gene ID	Base Mean	log_2_ Fold Change	Lfc SE	*P*-value (adjusted)	Cultivar	Timepoint	BLASTx description
TraesCS4A02G078200.1	127.4	−26.5	2.1	< 0.01	Stigg	6	hypothetical protein OsI_10614
TraesCS4D02G268900.1	24.2	−24.9	3.1	< 0.01	Stigg	6	predicted protein
TraesCS6A02G238300.1	54.6	−30	2	< 0.01	Stigg	6	No blast hit
TraesCS6B02G361500.1	13.3	−28	3	< 0.01	Stigg	6	No blast hit
TraesCS7D02G382200.2	49.5	−28.9	3.3	< 0.01	Stigg	6	No blast hit
TraesCS1D02G180900.1	48.8	18.6	4.2	< 0.01	Stigg	6	No blast hit
TraesCS2D02G346900.2	42.8	19	4.2	< 0.01	Stigg	6	No blast hit
TraesCS3A02G056800.1	28.7	18.1	3.5	< 0.01	Stigg	6	No blast hit
TraesCS5B02G262100.1	52.3	18.4	3.5	< 0.01	Stigg	6	No blast hit
TraesCS5D02G009100.1	101.6	19.4	4.2	< 0.01	Stigg	6	No blast hit
TraesCS1A02G370600.1	45.9	− 44.9	2.8	< 0.01	Stigg	24	No blast hit
TraesCS1D02G376500.1	20.5	−44.1	2.8	< 0.01	Stigg	24	No blast hit
TraesCS2D02G315600.2	43.5	−44	4.2	< 0.01	Stigg	24	esterase AGAP003155
TraesCS3B02G238800.7	26.2	−45.8	3.6	< 0.01	Stigg	24	No blast hit
TraesCS5B02G262100.1	52.3	−45.8	3.5	< 0.01	Stigg	24	No blast hit
TraesCS1D02G010000.1	9.4	43.4	3.3	< 0.01	Stigg	24	serine/threonine-protein kinase 19 isoform X1
TraesCS3D02G457200.1	15.2	44.5	3.7	< 0.01	Stigg	24	No blast hit
TraesCS4D02G063500.1	21.8	43.6	2.9	< 0.01	Stigg	24	predicted protein
TraesCS4D02G229200.2	19.4	46.5	3.1	< 0.01	Stigg	24	triadin-like isoform X2
TraesCS5D02G130200.2	14.3	46.8	3.7	< 0.01	Stigg	24	No blast hit
TraesCS1A02G378500.1	13.8	45.6	2	< 0.01	Stigg	48	predicted protein
TraesCS3B02G565500.1	63	45.7	3.3	< 0.01	Stigg	48	predicted protein
TraesCS3D02G418400.6	110.7	47.2	3	< 0.01	Stigg	48	No blast hit
TraesCS5D02G235500.1	13.2	44.9	2.4	< 0.01	Stigg	48	predicted protein
TraesCS5D02G491700.1	16.1	44.8	2.9	< 0.01	Stigg	48	No blast hit
TraesCS2A02G283600.1	11.9	− 43.7	5.1	< 0.01	Stigg	48	No blast hit
TraesCS2B02G562200.1	32.4	−44.9	2.9	< 0.01	Stigg	48	predicted protein
TraesCS5D02G091800.1	12.4	−43.1	4.4	< 0.01	Stigg	48	ROTUNDIFOLIA like 8
TraesCS5D02G130200.2	14.3	−43.5	3.7	< 0.01	Stigg	48	No blast hit
TraesCS7D02G135800.3	13	−46.6	3.6	< 0.01	Stigg	48	No blast hit
TraesCS1D02G010000.1	9.4	44.8	3.3	< 0.01	Stigg	96	serine/threonine-protein kinase 19 isoform X1
TraesCS3D02G298100.1	18.4	47.2	3.7	< 0.01	Stigg	96	No blast hit
TraesCS4D02G091100.1	9.6	44.7	5.9	< 0.01	Stigg	96	hypothetical protein TRIUR3_06363
TraesCS5D02G091800.1	12.4	44.4	4.4	< 0.01	Stigg	96	ROTUNDIFOLIA like 8
TraesCS5D02G457200.1	18.3	44.3	4.9	< 0.01	Stigg	96	No blast hit
TraesCS2A02G460500.1	14.9	−45.6	4.3	< 0.01	Stigg	96	predicted protein
TraesCS3A02G056800.1	28.7	− 47.5	3.5	< 0.01	Stigg	96	No blast hit
TraesCS3A02G395000.2	12.8	−44.2	5	< 0.01	Stigg	96	Transcription factor bHLH128
TraesCS3D02G010700.1	34.2	−48.4	3.6	< 0.01	Stigg	96	No blast hit
TraesCSU02G049500.1	17	−43	7.2	< 0.01	Stigg	96	No blast hit
TraesCS2A02G283600.1	11.9	44.6	5.1	< 0.01	Longbow	6	No blast hit
TraesCS3A02G087800.2	23.2	44	2.9	< 0.01	Longbow	6	No blast hit
TraesCS3D02G457200.1	15.2	44.8	3.7	< 0.01	Longbow	6	No blast hit
TraesCS4D02G063500.1	21.8	44.5	2.9	< 0.01	Longbow	6	predicted protein
TraesCS7B02G425800.1	37.9	46.3	2.7	< 0.01	Longbow	6	predicted protein
TraesCS2D02G580700.1	2.9	−42.9	2.4	< 0.01	Longbow	6	No blast hit
TraesCS3D02G214100.3	27.2	−44.8	4.4	< 0.01	Longbow	6	No blast hit
TraesCS5D02G130200.2	14.3	−46	3.7	< 0.01	Longbow	6	No blast hit
TraesCS6A02G321100.1	32.1	−43.8	4.1	< 0.01	Longbow	6	No blast hit
TraesCSU02G198000.1	18	−45.8	3.6	< 0.01	Longbow	6	Dirigent protein [Source:UniProtKB/TrEMBL;Acc:A0A341ZF53]
TraesCS2D02G059200.1	15.3	−45.4	4.7	< 0.01	Longbow	24	ribosome production factor 2 homolog
TraesCS3D02G214100.3	27.2	−46.4	4.4	< 0.01	Longbow	24	No blast hit
TraesCS4A02G175900.3	24.1	−45	4.6	< 0.01	Longbow	24	Glucan endo-1,3-beta-glucosidase [Source:UniProtKB/Swiss-Prot;Acc:P52409]
TraesCS4A02G403000.1	18.2	−44.3	3.8	< 0.01	Longbow	24	uncharacterized protein LOC100822466
TraesCS6B02G249700.1	15.5	−39.6	4.4	< 0.01	Longbow	24	GrpE protein homolog [Source:UniProtKB/TrEMBL;Acc:A0A1D6APT2]
TraesCS1A02G370600.1	45.9	47.8	2.8	< 0.01	Longbow	24	No blast hit
TraesCS1A02G370700.1	31.1	47.9	2.8	< 0.01	Longbow	24	No blast hit
TraesCS2D02G315600.2	43.5	48.2	4.2	< 0.01	Longbow	24	esterase AGAP003155
TraesCS5D02G009100.1	101.6	47.6	4.2	< 0.01	Longbow	24	No blast hit
TraesCS7A02G201300.1	17.7	47.5	2.8	< 0.01	Longbow	24	protein TIFY 11e-like
TraesCS1D02G389200.1	27.9	43.1	3.1	< 0.01	Longbow	48	predicted protein
TraesCS3A02G056800.1	28.7	44.6	3.5	< 0.01	Longbow	48	No blast hit
TraesCS3B02G565500.1	63	46.3	3.3	< 0.01	Longbow	48	predicted protein
TraesCS3D02G010700.1	34.2	45.8	3.6	< 0.01	Longbow	48	No blast hit
TraesCS5D02G130200.2	14.3	43.7	3.8	< 0.01	Longbow	48	No blast hit
TraesCS2D02G346900.2	42.8	−45	4.2	< 0.01	Longbow	48	No blast hit
TraesCS3D02G034200.1	17.9	−39.8	3.7	< 0.01	Longbow	48	No blast hit
TraesCS5B02G262100.1	52.3	−47.6	3.5	< 0.01	Longbow	48	No blast hit
TraesCS7B02G027400.1	7.8	−36.5	6.7	< 0.01	Longbow	48	F-box protein At5g67140 isoform X1
TraesCS7B02G160200.2	15.4	−43.7	4.3	< 0.01	Longbow	48	No blast hit
TraesCS2D02G059200.1	15.3	45.9	4.7	< 0.01	Longbow	96	ribosome production factor 2 homolog
TraesCS4D02G063500.1	21.8	47.4	2.9	< 0.01	Longbow	96	predicted protein
TraesCS4D02G091100.1	9.6	41.3	5.9	< 0.01	Longbow	96	hypothetical protein TRIUR3_06363
TraesCS5D02G491700.1	16.1	45.6	2.9	< 0.01	Longbow	96	No blast hit
TraesCS6A02G321100.1	32.1	45.8	4.1	< 0.01	Longbow	96	No blast hit
TraesCS2B02G495700.2	15.5	−47.1	3.6	< 0.01	Longbow	96	No blast hit
TraesCS2D02G346900.2	42.8	−48.1	4.2	< 0.01	Longbow	96	No blast hit
TraesCS3B02G238800.7	26.2	−43.5	3.6	< 0.01	Longbow	96	No blast hit
TraesCS4A02G175900.3	24.1	−44.7	4.6	< 0.01	Longbow	96	Glucan endo-1,3-beta-glucosidase [Source:UniProtKB/Swiss-Prot;Acc:P52409]
TraesCSU02G198000.1	18	−43.3	3.6	< 0.01	Longbow	96	Dirigent protein [Source:UniProtKB/TrEMBL;Acc:A0A341ZF53]

### The transcriptional response to *Z. tritici* differs between cultivars

To characterise the response of each cultivar at each timepoint, DEGs were mapped and annotated with Blast2Go (Additional file 3), and subsequently assigned to a biological process. The biological processes for each cultivar x timepoint combination were then categorised into 12 high-level groups based on the general role of the biological processes. These categories were: biosynthesis, catabolism, growth/development, hormone response, metabolism, photosynthesis, post-translational modification, oxidative stress, stress response, transcription, transport, and other. The number of DEGs within these categories that were down and up-regulated at in each cultivar was counted and the two cultivars were compared for their response across these categories. For each of these categories, we mined a publicly-available microarray study of susceptible versus resistant wheat cultivars (Gallant and Stigg, respectively) treated with *Z. tritici* [25] for independent validation of differential expression of genes involved in these key processes (Additional file 4). Where genes were differentially expressed in both datasets we have made a comparison between them. Where no comparison is made, the gene in question was not differentially expressed in the microarray data. In this microarray study, the susceptible cv. Gallant developed symptoms of STB by 10 days post inoculation, and had over 25% leaf area bearing pycnidia by 28 days post inoculation. This is a similar to cv. Longbow, which, in this experiment, displayed 20% leaf area bearing pycnidia at 28 dpi. Similarly in both experiments the resistant cv. Stigg showed < 5% leaf area bearing pycnidia in the Microarray study, and 1.5% leaf area bearing pycnidia in this study. In cv. Gallant, as in cv. Longbow, the main biological processes that were differentially expressed were the oxidation reduction process, the stress response, post translational modification and the regulation of transcription.

**Fig. 3:**
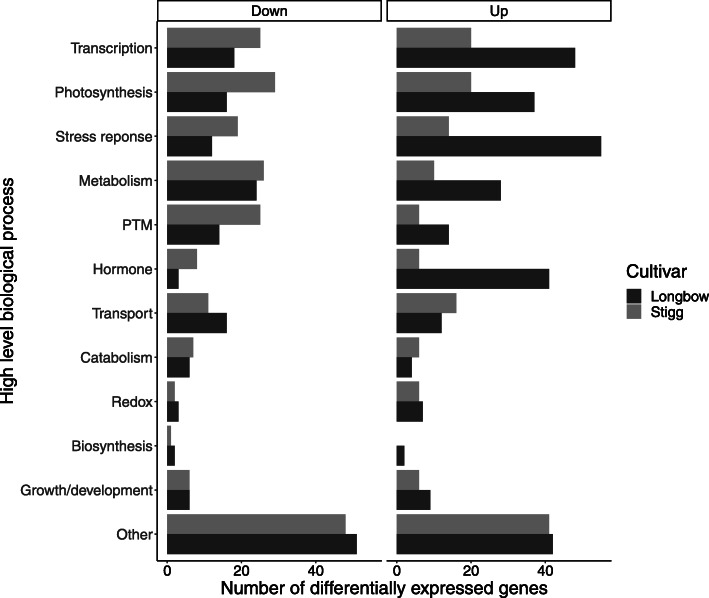
The most dominant biological processes in the differentially expressed genes were categorised into 12 high-level groups. Biological processes differentially expressed in Longbow are in red, and those in Stigg are in teal. The top panel shows down-regulated processes, the bottom panel shows up-regulated processes

In general, there are more up-regulated processes in cv. Longbow compared to cv. Stigg. In fact, of the DEGs involved in the most dominant biological processes, there are more down-regulated genes from cv. Stigg than cv. Longbow (147 versus 117), and more up-regulated genes in cv. Longbow than cv. Stigg (160 versus 110) (Fig. 3).

Cultivars Stigg and Longbow both responded to STB infection with the regulation of genes involved in post-translation modification (PTM), specifically those involved in protein phosphorylation, dephosphorylation, glycosylation and protein ubiquination. Across all timepoints, there appears to be higher expression of PTM-involved genes in cv. Longbow, suggesting a greater level of PTM happening in the plant in response to stress. Genes involved in protein phosphorylation (PP) were down-regulated at all 4 timepoints in cv. Stigg, but only at 6 hpi in Longbow, indicating active suppression of this type of PTM in cv. Stigg. In terms of up-regulated protein phosphorylation genes, three genes, all with protein kinase activity, were up-regulated at 6 h uniquely in cv. Stigg, TraesCS3B02G238800.7, TraesCS3B02G424200.3, and TraesCS5B02G528300.2. One of the kinase genes, TraesCS3B02G238800.7, was up-regulated in cv. Stigg at 6 h, then down-regulated at 24 h. This expression pattern was mirrored in cv. Longbow but with a time-delay; the gene was up-regulated in cv. Longbow at 24 h, then down-regulated at 96 h. The other two genes, TraesCS3B02G424200.3, and TraesCS5B02G528300.2, were not differentially expressed in cv. Longbow. By contrast, there is a large peak of 8 up-regulated PP genes at 24 h in Longbow, but no PP genes were up-regulated in Stigg at this timepoint.

Protein ubiquination, a second type of post-translational modification, was down-regulated in cv. Stigg from 6 h post inoculation. This down-regulation continued through to 48 h post inoculation. However, in cv. Longbow, the downregulation of protein ubiquination did not start until 24 hpi and was to a lesser extent than in cv. Stigg. That said, the downregulation of the ubiquitination process continued in cv. Longbow at 96 h post inoculation, by which point it had stopped in cv. Stigg. No genes involved in the ubiquitination process were up-regulated in either cultivar. The data suggest a slight time-lag in the down-regulation of PTM-related genes in cv. Longbow compared to cv. Stigg, and an overall reduction in activity of PTM-related genes in cv. Stigg. One of the PTM-related genes, a gene involved in protein glycosylation (TraesCS1A02G361100.1) was up-regulated in cv. Longbow at 96 h (log2 fold change = 1.2) and was also up-regulated in the STB-susceptible cv. Gallant at 24 hpi with *Z. tritici*, based on an independent microarray study (log2 fold change = 1.7) [25].

**Fig. 4:**
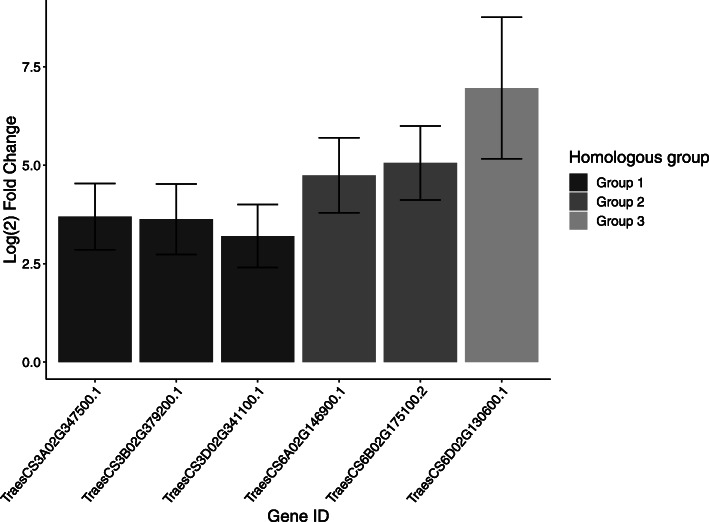
Six hormone-responsive genes were up-regulated in the STB-susceptible cv. Longbow at 24 h. Five genes fell into two homoeologous groups, that were identified as putative WRKY-like transcription factors (groups 1 and 2). The gene in group 3 was a putative SAUR7-like Auxin responsive gene. None of these genes were differentially expressed in cv. Stigg, nor at any timepoint other than 24 hpi in Longbow. Bars indicate SEM of the log_2_ fold change

In the susceptible cv. Longbow, biological processes involved in response to plant hormones, and hormone-responsive signalling pathways are up-regulated. At 6, 24, and 48 h, transcripts involved in the response to auxin, jasmonic acid, salicylic acid, abscisic acid and gibberellic acid were up-regulated in cv. Longbow, whereas the up-regulation of these processes is almost completely absent in cv. Stigg (only 2 genes, both predicted TIFY-like transcription factors, were differentially expressed in cv. Stigg). At 24 hpi there is a peak in hormone-responsive transcripts up-regulated in cv. Longbow, with 6 up-regulated hormone-responsive genes up-regulated that are not differentially expressed in cv. Stigg (Fig. 4; Additional file 5). Five of these transcripts fall into two homoeologous groups; three on the group three chromosomes, and two on the group 6 chromosomes, and the 6th is on chromosome 6D but is not part of homoeologous group. The group 3 hormone responsive genes are putative WRKY transcription factor-like based on their high homology to WRKY33 genes from *T. urartu* and *Aegilops tauschii*, and all three homoeologues are up-regulated in Longbow at 24 h*.* The group 6 hormone responsive genes are also putative WRKY transcription factor- like genes, although only the A and B copies are up-regulated in Longbow at 24 h. Two of these WRKY-like genes, TraesCS3A02G347500.1 and TraesCS6B02G175100.2, are also up-regulated in the STB-susceptible cv. Gallant at 24 hpi with *Z. tritici* based on the microarray study (Log_2_ fold change = 1.3 and 2.1, respectively).

The gene on chromosome 6D is a putative SAUR71-like Auxin responsive protein. A further observation from the hormone responsive genes was that in cv. Longbow, the response did not go beyond 24 h post-inoculation, i.e. no hormone-related genes were differentially expressed at either 48- or 96-h post-inoculation. However, two genes were differentially expressed in cv. Stigg at 48 hpi, both predicted TIFY-11e like transcription factors. TraesCS7A02G201300.1 was up-regulated in cv. Longbow and down-regulated in cv. Stigg at 24 h and was subsequently up-regulated in cv. Stigg at 48 h. The second TIFY-11e like gene, TraesCS7D02G204700.1, was up-regulated in both cvs. Stigg and Longbow at 24 h, and also up-regulated in cv. Stigg at 48 h. None of the hormone-responsive genes were differentially expressed at 96 hpi in either cultivar, suggesting that these genes are specific to a very early response to STB infection.

The regulation of genes involved in photosynthesis (the biological processes in this category are photosynthesis, electron transport chain and oxidation-reduction) shows an earlier response to the pathogen in cv. Stigg, compared to cv. Longbow. Furthermore, cv. Stigg displayed a more active response in terms of down regulating these biological processes. Across the majority of the timepoints, the oxidation-reduction (OR) process was the most dominant biological process in the DEGs. At 6 h post-inoculation, the number of up-regulated genes involved in the oxidation-reduction process was higher in cv. Longbow [5] than in cv. Stigg [3], and the number of down-regulated oxidation-reduction genes was higher in cv. Stigg [6] than in cv. Longbow [4]. The number of oxidation-reduction genes up-regulated in cv. Longbow increased at 24 hpi to 11 genes versus 2 in cv. Stigg. This number increased again at 48 h to 6 genes up-regulated in cv. Stigg, showing an opposite fluctuation to the pattern observed in cv. Longbow. In addition to the oxidation-reduction process being less up-regulated in cv. Stigg compared to cv. Longbow, more genes involved in this process are actively down-regulated in cv. Stigg, compared to cv. Longbow. By 96 hpi there are no oxidation-reduction genes down-regulated in cv. Longbow, compared to 6 down-regulated in cv. Stigg. TraesCS2A02G438200.1, a putative Ubiquinol oxidase gene that is involved with photosynthesis, was up-regulated in cv. Longbow at 48 h (log fold change =3.5) and in cv. Gallant at 24 h in the microarray study (log_2_ fold change = 3.7).

The category ‘Stress response” included genes annotated with the biological processes “defence response” (to any biotic or abiotic stress), “regulation of defence response”, or “pathogenesis”. Across the data, cv. Longbow showed a larger upregulation of stress responsive genes than cv. Stigg; 7 genes were up-regulated in cv. Longbow versus 1 in cv. Stigg. At 24 h in cv. Longbow two homoeologous Peroxidase genes, TraesCS2A02G107600.1 and TraesCS2B02G125300.1 were up-regulated, and were not differentially expressed in Stigg. One of these peroxidase genes, TraesCS2A02G107600.1, was also up-regulated by *Z. tritici* in cv. Gallant at 8 days post inoculation based on the microarray data (log_2_ fold change = 4.5). A third putative peroxidase gene, TraesCS6A02G047200.1, was up-regulated in cv. Stigg at 96 h, and was the only “stress response” genes to be up-regulated in cv. Stigg during the time points examined. TraesCS3A02G354800.1, a putative NPR4 gene was up-regulated in cv. Longbow at 96 h (log2 fold change = 1.77) and cv. Gallant at 24 h in the microarray study (log2 fold change = 2.2).

### Cultivar-specific responses to *Z. tritici* infection

**Fig. 5:**
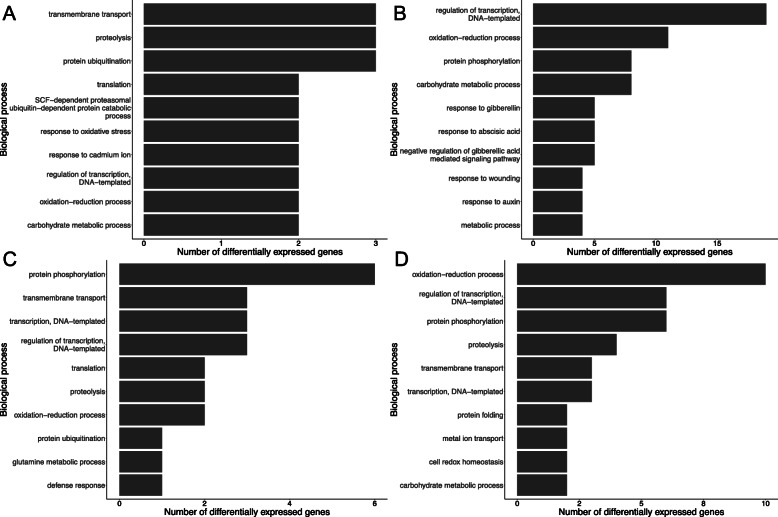
The top biological processes represented in the differentially expressed genes. **a** DEGs up-regulated only in cv. Longbow, **b** DEGs down-regulated only in cv. Longbow **c** DEGs down-regulated only in cv. Stigg D) DEGs up-regulated only in cv. Stigg

**Fig. 6:**
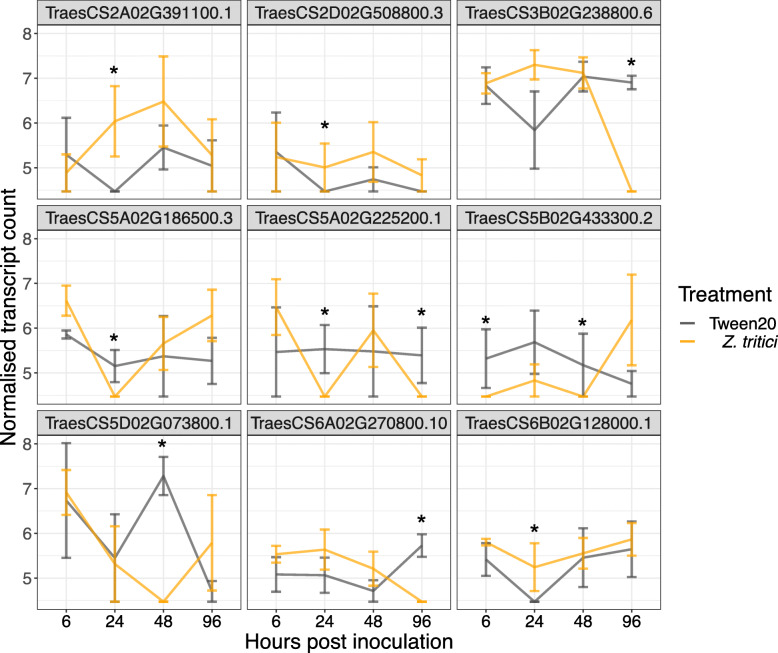
Gene expression, in cv. Stigg, of differentially expressed genes involved in protein phosphorylation (top two rows) and transmembrane transport (bottom row). These genes were differentially expressed in cv. Stigg but not in cv. Longbow. Asterisks show the timepoint at which the gene is differentially expressed in cv. Stigg. Gene expression is shown as the mean normalised transcript count across the three trials (via a variance stabilising transformation). Error bars = SEM

In addition to exploring the general response of each cultivar to *Z. tritici* infection, the DEGs that were unique to each cultivar were also examined and gene ontology analysis was conducted. In cv. Longbow, the most abundant down-regulated biological processes were transmembrane transport, proteolysis and protein ubiquination and the most abundant up-regulated processes were the regulation of transcription, oxidation reduction and protein phosphorylation. In cv. Stigg, protein phosphorylation was the most common down-regulated processes, followed by transmembrane transport and transcription. The oxidation reduction process was the most common up-regulated process, followed by the regulation of transcription and protein phosphorylation. In terms of the down-regulated biological processes, the most striking differences between the two cultivars were in the categories protein phosphorylation and transmembrane transport (Fig. 5). Six genes involved in protein phosphorylation were down-regulated in cv. Stigg, whereas no genes involved in PP were down-regulated in cv. Longbow. These genes are all predicted kinases: TraesCS3B02G238800.6 and TraesCS5B02G433300.2 are predicted casein kinase 1-like proteins, TraesCS5A02G186500.3 is a predicted LEMK1.1 protein, TraesCS5A02G225200.1 is a predicted CBL-interacting protein kinase, TraesCS5D02G073800.1 is a predicted wall-associated receptor kinase-like 14 and TraesCS6A02G270800.10 is a predicted Serine/threonine protein kinase ppk15-like (based on NCBI BLASTn). These genes were uniquely down-regulated in cv. Stigg; they were not up-regulated in cv. Stigg or differentially expressed in cv. Longbow at any of the timepoints examined. In transmembrane transport, at 24 hpi, three transcripts involved in transport were up-regulated in cv. Stigg that were not differentially expressed anywhere else in the data. These genes had predicted transporter functions based on homology to *Aegilops tauschii*. TraesCS2A02G391100.1 is a predicted metal-nicotianamine transporter YSL9, TraesCS2D02G508800.3 is a probable sulphate transporter 3.3, and TraesCS6B02G128000.1 is a putative zinc transporter 2-like (Fig. 6).

Aside from protein phosphorylation and transmembrane transport, there were no large differences in the most abundant GO categories of the cultivar-specific DEGs. Therefore, to explore further cv. Stigg-specific responses, the ‘Other’ category of high-level biological processes was expanded, and specific GO terms that were present in cv. Stigg and not in cv. Longbow were identified. Eight GO terms were associated with 5 Stigg-specific DEGs, and were not present in the cv. Longbow DEGs. One of these genes down-regulated in cv. Stigg is involved in response to red or far red light. The remaining four genes were all up-regulated and are involved in iron-sulphur cluster assembly, RNA processing and protein folding.

### Common transcriptional changes between Stigg and longbow

**Fig. 7:**
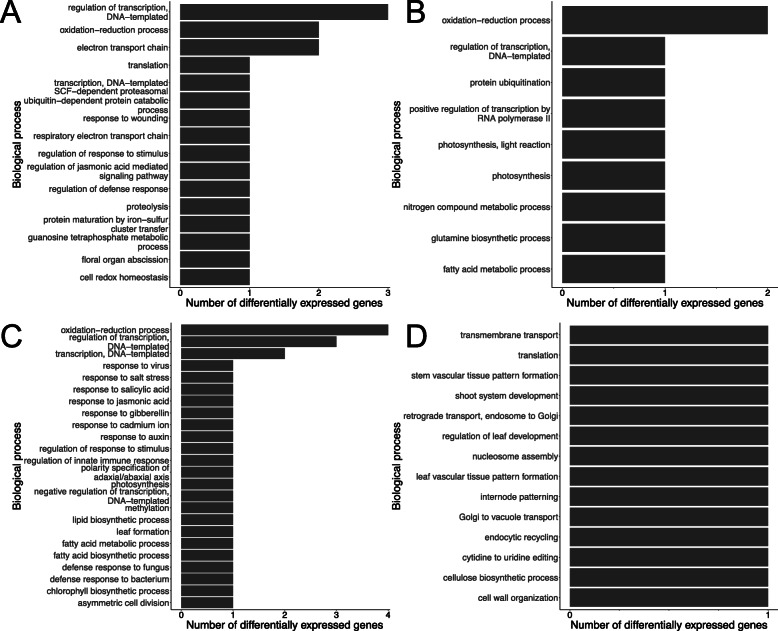
The top 10 biological processes represented within the genes that were differentially expressed in both cvs. Stigg and Longbow. The genes were divided into 4 subsets: **a** Longbow up-regulated and Stigg up-regulated, **b** Longbow down-regulated and Stigg down-regulated, **c** Longbow up-regulated and Stigg down-regulated, and **d** Longbow down-regulated and Stigg up-regulated

In addition to cultivar-specific responses to *Z. tritici*, we explored the biological processes that were differentially expressed across both cultivars (Fig. 7). In total, 160 gene were differentially expressed in both cultivars. These DEGs were separated into four subsets: ‘Longbow up and Stigg up’, ‘Longbow down and Stigg down,’ ‘Longbow up and Stigg down’, and ‘Longbow down and Stigg up’. In the categories ‘Longbow up Stigg up’, ‘Longbow down Stigg down’ and ‘Longbow up Stigg down’, the major biological processes were the regulation of transcription and the oxidation-reduction process. In the category ‘Longbow up Stigg down’, there are many biological processes associated with the defence response, including the previously mentioned hormone response processes, the regulation of response to stimulus and defence response. Therefore it seems that not only are genes within these categories up-regulated in cv. Longbow and not up-regulated in cv. Stigg (as discussed earlier), there are genes in these categories that are up-regulated in cv. Longbow and down-regulated in cv. Stigg. In the category ‘Longbow down Stigg up’ there are biological processes involved in growth and development, for example stem vascular tissue pattern formation, shoot system development, and the regulation of leaf development.

### Gene ontology enrichment analysis

**Fig. 8:**
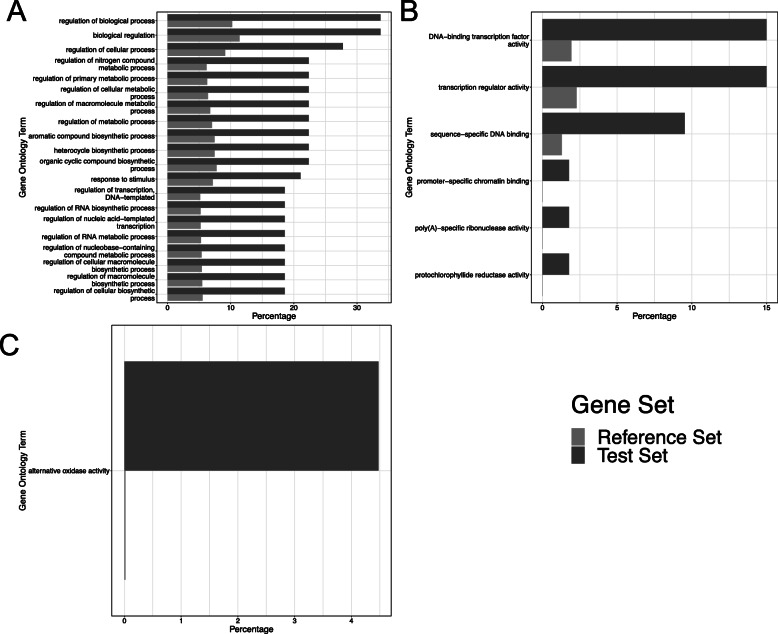
GO enrichment analysis revealed over represented gene ontology terms in genes that were up-regulated in cv. Longbow at 24 and 48 hpi with *Z. tritici.* **a** the top 20 over-represented biological processes in cv. Longbow, 24 hpi, up-regulated **b** the 6 over-represented molecular functions in cv. Longbow, 24 hpi, up-regulated **c** At 48 h, 1 biological process was overrepresented in cv. Longbow, 24 hpi, up-regulated

Gene ontology enrichment analysis was performed on all subsets of the data (i.e. for both cultivars at all 4 timepoints, separated by down and up-regulated genes). Differentially expressed gene sets were compared to the set of all genes expressed across the data to identify over or under represented GO categories. In cv. Longbow, at 24 and 48 h post inoculation, over represented GO terms were identified (Fig. 8). No over-represented GO terms were found in any of the cv. Stigg data sets. At 24 hpi in cv. Longbow, 84 biological processes and 6 molecular functions were over represented in the up-regulated genes. The most over represented GO terms were the regulation of biological processes, cellular processes, metabolic processes and biosynthetic processes. The GO term ‘response to stimulus’ was also over-represented. Over-represented molecular functions include transcription factor activity, transcription regulator activity, DNA binding and chromatin binding. At 48 hpi in cv. Longbow, one molecular function was over represented, ‘alternative oxidase activity’.

In general, it appears that although there are many similarities in the response to *Z. tritici* between cvs. Stigg and Longbow, there are some clear differences in both the numbers of genes from different biological processes that are differentially expressed, and also the direction of their expression (up versus down). As discussed, cv. Longbow displays more evidence of a stress response than cv. Stigg, with the upregulation of genes involved in peroxidase activity, pathogenesis and regulation of defence response, whereas only one gene from this category was up-regulated in cv. Stigg. Similarly, the response of genes involved in plant hormones was higher in cv. Longbow than in cv. Stigg, with genes involved in auxin, jasmonic acid, salicylic acid, abscisic acid, and gibberellic acid signalling all up-regulated in cv. Longbow with little activity in Stigg. Additionally, it was observed that some of the genes from these stress-response related biological processes that were up-regulated in cv. Longbow were also down-regulated in cv. Stigg. However, there are some evident cv. Stigg-specific responses, such as the down-regulation of genes involved in protein phosphorylation that is not evident in cv. Longbow, and the upregulation of transmembrane transport. Additionally in cv. Stigg, there is little evidence of the hormone-mediated defence pathways, response to oxidative stress, and transcription of defence genes that were observed in cv. Longbow. It seems, therefore, that while both cvs. Longbow and Stigg response to *Z. tritici* infection as early as 6 hpi with altered transcription of a range of genes, there are many cultivar-specific transcriptional response that may warrant further investigation in the elucidation of cv. Stigg’s exceptional resistance to STB disease.

### Weighted gene co-expression network analysis

To identify modules of co-expressed genes and identify potential interactants of the STB-responsive genes identified by differential expression analysis, expression matrices of *T. aestivum* and *Z. tritici* genes across all samples were analysed with the “WGCNA” package in R. Networks were constructed separately for cvs. Stigg and Longbow to identify and delineate both shared and cultivar specific networks of co-expression. A total of 94,707 genes from both the *T. aestivum* and *Z. tritici* genomes were assigned to modules based on their co-expression form the RNAseq data. Within the cv. Longbow network, 185 sub-networks, or ‘modules’ were identified (Additional file 6), and within the cv. Stigg network 209 modules were identified (Additional file 7).

Within the 185 modules in the cv. Longbow co-expression network, six modules had a significant correlation with treatment: modules L3 (C_c_ = 0.68), L17 (C_c_ = 0.588), L31(C_c_ = 0.55), L103 (C_c_ = 0.50), L155 (C_c_ = 0.52) and L177 (C_c_ = 0.51). Within the cv. Stigg co-expression network, three cv. Stigg modules had a significant positive correlation with treatment, modules S2 (C_c_ = 0.62), S20 (C_c_ = 0.56), and S31 (C_c_ = 0.59). The hub genes (i.e. the most connected gene in each module) were identified and characterised by a BLASTn search to the NCBI non-redundant nucleotide database. All six of the cv. Longbow modules, and two of the cv. Stigg modules were comprised of a mixture of *T. aestivum* and *Z. tritici* genes (Table 3).

**Table 3:** Gene co-expression network modules that are significantly correlated with treatment with *Zymoseptoria tritici*

Dataset	Module	Number of genes		C_c_ (Treatment)	Hub gene	BLASTn Top Hit
		*T. aestivum*	*Z. tritici*			
Longbow	3	3329	3101	0.68	Mycgr3T99044	Glyceraldehyde-3-phosphate dehydrogenase
	17	904	1	0.59	TraesCS5B02G056700.1	L-type lectin-domain containing receptor kinase
	31	438	19	0.56	TraesCS7A02G198300.1	probable galacturonosyltransferase-like 9
	155	44	39	0.53	Mycgr3T71062	Hypothetical protein
	177	27	28	0.52	Mycgr3T83303	putative major facilitator superfamily transporter
	103	59	86	0.5	Mycgr3T47614	Hypothetical protein
Stigg	2	3739	3305	0.63	Mycgr3T44978	Hypothetical protein
	20	710	0	0.56	TraesCS5D02G472000.1	Probable inorganic phosphate transporter 1–8
	31	266	172	0.59	Mycgr3T109435	Proteoglycan-like protein

C_c_ is the correlation coefficient of the module to treatment. Hub genes are the most connected gene within the network module, which were annotated by BLASTn search to the NCBI non-redundant nucleotide database. Gene IDs that start with ‘Mycgr’ are *Z tritici* genes. Genes that start with ‘Traes’ are *Triticum aestivum* genes

**Fig. 9:**
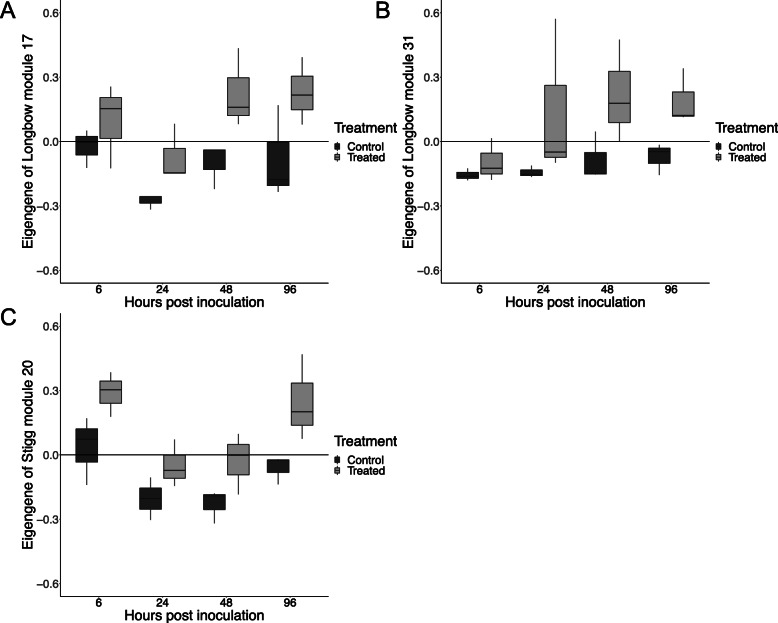
The module eigengene (first principle component of a co-expression module) of cv. Longbow modules L17 (**a**) and L31 (**b**), and cv. Stigg module S20 (**c**) were significantly higher in the treated samples than the control samples, suggesting that average expression of the genes in these modules is higher in response to *Z. tritici* compared to Tween20

For cv. Longbow modules L17, L31 and cv. Stigg module S20, which were populated by a majority of wheat genes, the module eigengenes (the first principle component of the module) were significantly higher in the treated samples than the control (Kruskal-Wallis *P*-value < 0.05; Fig. 9). The differentially expressed genes within the modules that were significantly correlated with treatment and comprised mostly *T.* aestivum genes were counted. In general, these modules contained more genes that were pathogen-responsive in cv. Longbow than in cv. Stigg, including the cv. Stigg module S20. The presence of differentially expressed genes in these modules indicate that the gene networks correctly cluster genes that are pathogen responsive. Of the 31 DEGs in module L17, 18 were pathogen down -regulated in cv. Longbow, 7 in cv. Stigg, and 4 in both cultivars. Two genes were pathogen up-regulated in cv. Longbow. L31 comprised 38 differentially expressed genes, of which 19 were pathogen up-regulated in cv. Longbow, 14 in cv. Stigg and four in both cultivars. One gene was pathogen down-regulated, in cv. Stigg. The cv. Stigg module S20 contained 20 DEGs, 14 of which were pathogen up-regulated cv. Longbow, 6 were up-regulated in cv. Stigg, one was up-regulated in both cultivars and one was down-regulated in Longbow. The hub genes, TraesCS5B02G056700.1 (L17), TraesCS7A02G198300.1 (L31), and TraesCS5D02G472000.1 (S20) are not differentially expressed.

**Fig. 10:**
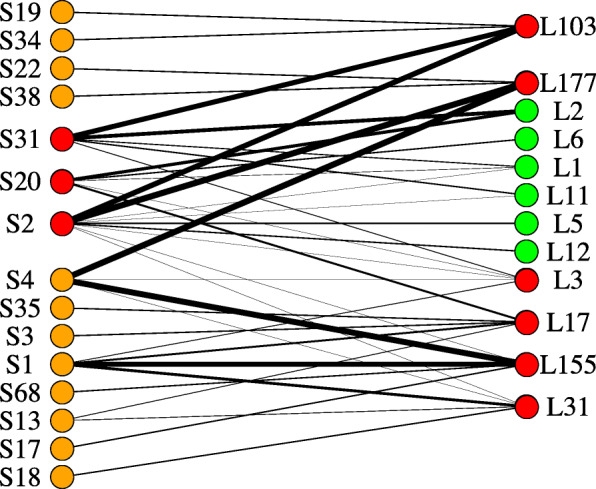
Module preservation between cv. Stigg (left side) and cv. Longbow (right side) co-expression modules that were correlated with treatment. Modules that have a significant correlated with treatment are shown with red nodes. Edges connect modules that share genes, and edge weight (i.e. line thickness) represents the number of genes shared between the modules

Module preservation was calculated between the two networks (cvs. Longbow and Stigg) to identify which modules were conserved between both cultivars, and which were cultivar specific. As per Langfelder and Horvath [26], modules with a preservation (Z) score > = 10 were considered preserved, those with a score between 2 and 10 were considered moderately preserved, and those with a score < 2 were considered not preserved between the data sets. Within the cv. Longbow set, the mean Z-score was 9.8. Of the 184 modules, 52 (24.7%) were well-preserved, 66 (31.4%) were moderately preserved, and 68 (32.3%) were not preserved. The mean Z-score of the cv. Stigg modules was 8.7. Fifty-four (25.7%) modules were well-preserved, 54 (25.7%) were moderately preserved, and 102 (48.5%) were not preserved. All six of the cv. Longbow modules that correlated with treatment (modules L3 (C_c_ = 0.68), L17 (C_c_ = 0.588), L31 (C_c_ = 0.55), L103 (C_c_ = 0.50), L155 (C_c_ = 0.52) and L177 (C_c_ = 0.51)) were preserved in the cv. Stigg set: 5 of them were well preserved and one was moderately preserved. All three of the cv. Stigg modules that correlated with treatment (S2 (C_c_ = 0.62), S20 (C_c_ = 0.56), and S31 (C_c_ = 0.59)) were well-preserved in the cv. Longbow set. For each preserved module, the corresponding modules in the opposite data set were identified (i.e. the modules that contain that same genes as the reference module) (Fig. 10). In general, the modules from both cultivars that were correlated with treatment were well preserved with each other, suggesting that the genes within these modules have a similar response to *Z. tritici.* However, module L3 (from cv. Longbow) did not have strong connections with cv. Stigg modules that were also correlated with treatment, suggesting that the genes within this module have expression patterns more specific to cv. Longbow than to cv. Stigg. The dominant biological process in this module was protein phosphorylation (352 genes), followed by oxidation-reduction (166 genes) and the regulation of transcription (137 genes). The top three molecular functions represented in this module were ATP-binding, protein binding, and protein kinase activity.

## Discussion

*Z. tritici* is a serious threat to wheat production across Europe, and as such, elucidating the molecular response to *Z. tritici* is paramount for gene identification and for informing breeding practice. In this study, we characterized the genetic response to *Z. tritici* in both an STB-resistant and an STB susceptible wheat cultivar. The generation of 60.9 million reads per sample equated to ~ 25 X coverage of the complete wheat and *Z. tritici* transcriptome (~ 240 million bases in the combined transcriptome) and meant that detection of *Z. tritici* genes was possible. On average, 2554 *Z. tritici* genes were detected in the treated samples, representing 1.7% of the total mapped reads. It is possible that some level of background contamination occurred as all the plants were grown in the same growth room, however these pathogen genes that are mapped in the control samples may be due to mis-alignment of conserved genes. The number of pathogen genes detected represents 23% of the *Z. tritici* genome, a third as many as detected by Rudd et al., (2015). However, the percentage of total reads mapped to the *Z. tritici* genome is similar: an average of 1.4% of the total reads were mapped to the *Z. tritici* reference in the first 4 days of infection, at a depth of coverage of 45 million reads per sample [27], compared to 1.7% of reads mapped to the pathogen out of the 60 million reads per sample in this study. Additionally, the number of *Z. tritici* reads mapped in this study is more than the number of pathogen reads detected in a dual-RNAseq study of the powdery mildew pathogen *Erysiphe pisi* on its *Medicago trunculata* host [28], where 0.5% of the detected reads belonged to the pathogen, at a depth of coverage of 95 million reads per sample (compared to 60 million reads per sample in this study). Ongoing dual-RNAseq studies of a Stigg x Longbow segregating population is being conducted in our laboratory to delineate the association between specific host genes and the inheritance of STB resistance; this study, together with the outputs of the study herein, will also provide a comprehensive pathogen transcriptome dataset to mine for information about the pathogens response in these wheat hosts.

Differential expression analysis, comparing treated versus control samples within each genotype x timepoint combination revealed ~ 500 high-confidence wheat genes that were differentially expressed in response to treatment. Given the chromosomal distribution of the total number of expressed genes, in which each genome (A, B and D) contributed equal numbers of expressed genes, we would expect a ratio of 32:32:32% of differentially expressed genes across the experiment (the remaining 4% of genes are not assigned to a chromosome). The ratio of DEGs across both cultivars did not deviate from this ratio, however when individual datasets were examined, we observed a significant deviation from the expected ratio in the transcripts that were up-regulated in cv. Stigg. This result suggested some evidence of sub-genome bias in cv. Stigg’s response to *Z. tritici*; an increase in gene transcripts from the A genome and a lower than expected numbers of DEG’s came from the B genome. Sub genome bias is a well-documented phenomenon in polyploid plant species and can result from buffering of functionally redundant homoeologues, or genetic dominance resulting from homoeologue-specific variation [29, 30]. More specifically, sub-genome bias has been shown to be especially prevalent with respect to the plants response to stress; for example in wheat infected with the fungal pathogen *Fusarium pseudograminearum*, the response of the B and D genomes was greater than that of the A genome [31]. In addition to an overall bias in the stress response of cv. Stigg, we saw a timepoint-specific bias with a decrease in A-genome DEGs and an increase in B-genome DEGs at 6 h and 48 h post inoculation. Cv. Stigg (pedigree ((Biscay x LW96–2930) x Tanker) is derived from a synthetic hexaploid wheat (SHW) and contains at least 3 quantitative trait loci (QTL) for Septoria resistance, the strongest of which are on chromosomes 1B and 3B [10]. Synthetic hexaploid wheats (SHW) were originally created to introduce genetic diversity into breeding programmes by artificially generating fertile hybrids between the tetraploid (AABB) *Triticum turgidum* and the diploid (DD) goatgrass *Aegilops tauschii* [32]. Two types of synthetic hexaploids were created, between either modern tetraploid *T. turgidum* ssp. *durum* (genome AABB) and *A. tauschii* (genome DD), or wild tetraploid *T. turgidum* ssp. *dicoccoides* (AABB) and *A. tauschii* (DD) [33]. Cv. Stigg’s synthetic ancestor (LW96–2930) is derived from a *T. turgidum* subsp*. dicoccoides* derived SHW (S. Berry, personal comm.). Therefore, compared to many SHW’s that contain predominantly D-genome genetic variation, LW96–2930 provides genetic variation across all three sub-genomes. This SHW is a Septoria-resistant line and therefore is assumed to contribute a large proportion of cv. Stigg’s exceptional STB resistance and may explain the sub-genome bias observed in cv. Stigg as compared to cv. Longbow. The decrease in B-genome derived response may be explained by presence of a synthetic-derived introgression, which may respond to the pathogen differently to how a homologous locus from a modern wheat would. Additionally, the wild introgression may have caused a decrease in alignment of RNAseq reads to the reference as the genome sequence at this locus may be divergent from cv. Chinese Spring, the wheat variety from which the reference sequence is derived [34]. With respect to identifying candidate genes for resistance, genetic mapping and surveying allelic diversity between cvs. Stigg and Longbow for the identification and delineation of chromosomal introgressions in cv. Stigg from this synthetic parent may help pinpoint specific loci and genes that contribute to resistance.

To characterize the ~ 500 differentially expressed genes, we assigned them to high-level biological processes to facilitate the identification of important mechanisms in the response to *Z. tritici* infection. The most general observation from these data was that the STB-susceptible cv. Longbow responds to *Z. tritici* infection with an early defence response that is not observed in cv. Stigg. In particular, 24 hpi appears to be an important timepoint for cv. Longbow; at this time the processes up-regulated suggest that cv. Longbow has recognized, and responded to, the pathogen. Eight genes involved in post-translational modification (PTM), a process that can influence downstream defence signalling [35], were up-regulated at this timepoint. With respect to pathogen attack in plants, PTM can be induced by a burst of reactive oxygen species (ROS) that coincides with pathogen recognition [36]. Supporting evidence for a pathogen-mediated ROS burst is the up-regulation of two peroxidases at 24 h in cv. Longbow. Peroxidases are enzymes that catalyse the generation of ROS in response to pathogen attack in plants [37]. This is a reaction that can also be stimulated by plant defence-related hormones, such as Salicylic and Jasmonic acid, known to accumulate in response to pathogen attack [38]. This suggests that there may be some hormone activity in cv. Longbow in response to *Z. tritici*. Corroborating this is the expression of two families of hormone responsive WRKY transcription factor-like genes and an auxin-responsive SAUR7-like gene. Expression of WRKY transcription factors can be induced by the plant hormones salicylic and jasmonic acid [39], and these transcription factors have been shown to be important for resistance against pathogens in *Arabidopsis thaliana* [40] and in rice *Oryza sativa* [41]. Although shown to increase resistance on other species, the upregulation of these WRKYs does not confer resistance to STB in cv. Longbow. It has been suggested that *Z. tritici* may benefit from, and can manipulate production of host ROS, in particular H_2_O_2_ [19, 23]. Therefore, an early ROS burst in cv. Longbow may inadvertently serve to benefit the pathogen, contributing to its asexual success by reducing the length of the latent phase (~ 12 days in cv. Longbow) and facilitating the switch to necrotrophy. In addition to the up-regulation of genes in cv. Longbow, there was also down-regulation of genes involved in many of the biological processes involved in the plant defence response, such as transcription, the stress response, photosynthesis and metabolism. This is supported by the results of Rudd et al., 2015 [27], who saw the up-regulation of *Z. tritici* effector genes during the first 4 days of STB infection in the susceptible cv. Riband, and the down-regulation of these plant defence-associated processes in the wheat host. The authors suggest that these plant processes may be supressed by the pathogen, and the potential for fungal interference in the plant’s transcriptome.

In contrast to cv. Longbow, cv. Stigg showed a less dramatic response to *Z. tritici*. In fact, a suppression of PTM genes, hormone-related transcription factors, and general defence response genes was observed at 24 hpi in cv. Stigg. It seems, therefore, that rather than early detection and activation of defence responses to combat the pathogen, cv. Stigg’s languid response to attack may be contributing to its long latent phase, actively disadvantaging the pathogen. The observed differences between cv. Stigg and cv. Longbow’s defence strategies support the idea that *Z. tritici* has evolved to manipulate host defences for its own success [17], and suggest that when it comes to fighting off STB disease, less is more. In fact, suppressing a response and avoiding a ROS burst may stave off the pathogen and increase the time until the disease progresses to the necrotrophic phase, during which the fungus can start to reproduce. Although the triggers for the switch from the latent phase to the necrotrophic phase are still unknown, and are to some extent dependable on the host [21], the fungus has some genetic sovereignty over the length of the latent phase, and the switch to necrotrophy. The chromosomal makeup of *Z. tritici* is 13 chromosomes, and up to 8 accessory/dispensable chromosomes that show presence/absence polymorphism between different isolates of the fungus [18]. The adaptive function of these accessory chromosomes has not been fully elucidated but their absence, in part, confers fitness to the pathogen in the form of an earlier switch to necrotrophy and subsequent increased numbers of pycnidia [21]. One explanation for such fungus-lead necrotrophy is the secretion of small, cysteine-rich proteins by the fungus, termed ‘effectors’ [42]. These effectors can induce cell death in the plant [43] and peak in expression around the time of the necrotrophic switch [19]. The efflux of effectors from the fungus can be in response to pathogen-associated molecular pattern (PAMP)-triggered immunity (PTI) [36]. In the case of Longbow and the *Z. tritici* strain used in this study, it appears that a PAMP-triggered response is occurring, but this leads to susceptibility. As is documented in biotrophs [36], necrotrophs [44], and hemibiotrophs [45], this susceptibility may be effector triggered, as plant pathogens secrete effector proteins to overcome or supress the PAMP-triggered response. Therefore, we suggest that the susceptibility observed in Longbow may be due to the fungus potentially manipulating the host response, as has been previous suggested for *Z. tritici* [17].

In the case of cv. Stigg, the evidence for a PAMP-triggered response or ROS burst is weak. A PAMP-triggered response relies on recognition of the pathogen, suggesting either a lack of recognition by cv. Stigg, or recognition and a subsequent downregulation of PAMP responses. As previously mentioned, cv. Stigg is synthetically derived and therefore contains introgressions from a wild wheat relative. Synthetic hexaploid wheat have previously been shown to have good resistance to STB [46] and therefore we hypothesise that these introgressions are responsible for the lack of recognition between cv. Stigg and the pathogen. *Z. tritici* is a wheat-specific pathogen that has co-evolved with domesticated wheat and may have started as an endophyte [18]. Specialisation of *Z. tritici* for wheat occurred during the early domestication of hexaploid and tetraploid wheats, around 10,000 years ago [47], during which time genetic diversity within these species was reduced [30]. As such, *Z. tritici* can infect only a few species from the *Triticum* genus [48], and some species, particularly *T. monococcum*, show resistance to STB, characterized by full arrest of the fungus post entry into the stomata [49]. Therefore, we hypothesise that a chromosomal introgression from a wild wheat relative in Stigg may have replaced a locus from modern wheat that is usually required for pathogen recognition.

To further investigate the transcriptomic response of cvs. Stigg and Longbow to STB infection, we constructed gene expression networks to cluster wheat and *Z. tritici* genes into modules of co-expressed genes. In both cultivars, modules that were correlated with treatment were well preserved, suggesting that many of the disease responsive genes are consistent between these cultivars. However, certain cv. Longbow modules that were correlated with treatment did not show a strong relationship with cv. Stigg modules that were also correlated with treatment. This suggests that although these modules are preserved between the cultivars, their function is not, indicating a cultivar-specific response of their constituent genes. This analysis further supports a cv. Longbow-specific defence response. Furthermore, there were more modules from cv. Longbow that were correlated with treatment than in cv. Stigg, further strengthening the evidence that Longbow is more active in its response to STB than cv. Stigg.

## Conclusions

In conclusion, we present evidence that the STB-susceptible cv. Longbow responds to *Z. tritici* infection with a PAMP-triggered response that ultimately leads to susceptibility. In comparison, in the STB-resistant cv. Stigg there are fewer PAMP-related up-regulated genes, and some evidence of the down-regulation of key biological processes. This approach possibly allows it to stave off disease progression and extends the latent phase of disease. We hypothesise that introgressions from Septoria-resistant wild wheat relatives may reduce the host response to the pathogen. Further investigation into the genome of cv. Stigg, the ongoing dual-RNAseq studies of a Stigg x Longbow segregating population, and studies on the physiology of the early stages of infection with *Z. tritici* in this cultivar may further reveal the mechanisms of defence and allow for delineating the exact loci responsible for cv. Stigg’s recognition (or lack thereof) of the pathogen.

## Methods

### Germplasm

The hexaploid winter wheat cultivars (cvs.) Longbow and Stigg were used in this study. Longbow (Pedigree: TJB-268-175/HOBBIT) was released in 1980 and is susceptible to STB [23]. Stigg (Pedigree: (BISCAY/LW-96-2930//TANKER) was released in 2010 and has high resistance to STB [22]. Germplasm for both cultivars was provided by Dr. Simon Berry of Limagrain Ltd., Norwich, UK. Seeds for both cultivars are available from the John Innes Centre SeedStor (https://www.seedstor.ac.uk), Longbow: Store code W4115, Stigg: Store code W10052.

### Fungal material and inoculum preparation

The *Z. tritici* isolate used in this study is Cork Cordiale 4. This is a field isolate collected from the wheat cv. Cordiale in County Cork, Ireland, in 2016. Glycerol stocks were provided by Thomas Welch and Stephen Kildea (Teagasc Crops Research Centre, Oak Park, Co. Carlow, Ireland). *Z. tritici* was cultured by inoculating potato dextrose agar (PDA) with 10 μl of the glycerol stock and incubating the Petri dishes under near-ultraviolet light for 7 days at 20 °C, 12:12 h light:dark cycle). Inoculum was prepared as described in Zhou et al.*,* (2020) [50], ,and adjusted to a solution of 1 × 10^6^ spores ml^− 1^ + 0.02% Tween20. (Fisher Bioreagents, USA). A 0.02% Tween20 solution was used to inoculate the control plants.

### Septoria tritici blotch experiment

Seeds of cvs. Stigg and Longbow were stratified for 3 days at 4 °C and germinated for 5 days at 19 °C on moist Whatman No. 1 filter paper (Whatman International LTD, UK) in Petri dishes wrapped in aluminium foil. Seedlings were potted into John Innes compost Number 2 (Westland Horticulture, UK) in 7 × 7 cm pots with two plants per pot. For each genotype x treatment x timepoint combination, three pots (6 plants) were grown for RNA sequence analysis per harvest timepoint and three for disease phenotyping. Pots were placed in plastic trays and placed in a growth chamber at 19 °C under a 16:8 light:dark regime with 90% humidity. Within the trays the position of pots containing plants for both genotypes and all timepoints were randomised, but treated and control plants were always placed in different trays to avoid contamination. Plants were watered with 1 l of water in the trays every 3 days. At emergence of the 4th leaf, the 3rd leaf of each plant was marked, and spray-inoculated with either 2 ml 1 × 10^6^ *Z. tritici* spores ml^− 1^ + 0.02% Tween20 or a control solution of 0.02% Tween20 (1 ml each per adaxial and abaxial surface). In the case of plants grown for RNA sequence analysis, at either 6, 24, 48 or 96-h post inoculation, the entire inoculated leaf was excised using sterile forceps and scissors. Spray inoculations were done at 10 am on the morning of inoculations, and tissue was taken at 6 pm the same evening for the 6 h timepoint, and 10 am the following day, the day after that, and 2 days after that for the 24, 48, and 96-h timepoints. Biological replicates (i.e. 6 leaves) were pooled into one 50 ml falcon tube, flash frozen in liquid nitrogen and stored at -80 °C. For the phenotyping plants, disease on the 3rd leaf was scored as a percentage of leaf area bearing necrosis and pycnidia at day 28 post inoculation (dpi). The STB experiment comprised three independent trials, which were conducted one after the other and did not overlap.

### RNA extractions and sequencing

Leaf tissue was ground in liquid nitrogen using a sterile pestle and mortar. Total RNA was extracted from the 100 mg of ground tissue with the TriZol protocol (Invitrogen, California, USA) following the manufacturers protocol. RNA pellets were washed twice with ice-cold 70% ethanol and resuspended in 40 μl nuclease-free sterile water. gDNA was digested using TURBODNase (Ambion, USA) and RNA samples were cleaned and concentrated using the RNeasy mini kit from Qiagen (Hilden, Germany). RNA quality and integrity were checked using a Biodrop μLITE (Biochrom Ltd., Cambridge, UK) and Bioanalyzer 2100 (Agilent, Santa Clara, California, USA). RNA concentration was adjusted to 100 ng/μl and 30 μl was transferred to a sterile 0.5 ml Eppendorf tube. In total 48 samples (2 genotypes × 2 treatments × 4 timepoints × 3 independent trials) were sent (on dry ice) to Beijing Genomics Institute, Hong Kong. The 48 RNA samples were barcoded and split across 16 lanes of an Illumina HiSeq 2500 (Illumina, San Diego, California, USA) and paired-end 100 base-pair reads were generated.

### Pre-processing of RNAseq data and mapping reads to the reference genomes

Reads were demultiplexed, adapters were trimmed and reads containing more than 5% unknown nucleotides and more than 30% bases of Phred score lower than 10 were removed. FastQC (http://www.bioinformatics.babraham.ac.uk/projects/fastqc) was used to generate quality metrics for each paired-end FASTQ file (48 samples with paired end reads x = 96 FASTQ files). The individual quality report files were collated into one summary report using MultiQC [51]. The IWGSC Refseq version 1.1 cDNA annotation [34] was accessed from the IWGSC URGI portal (https://urgi.versailles.inra.fr/download/iwgsc/IWGSC_RefSeq_Annotations/v1.1/). Every transcript for each gene was represented in the reference. The reference genome contains 137,056 genes and gene isoforms, which represent 110,790 high-confidence genes. The *Zymoseptoria tritici* MG2 reference cDNA annotation was retrieved from EnsemblFungi (http://fungi.ensembl.org/Zymoseptoria_tritici/Info/Annotation). Two reference index files (*T. aestivum* only and a combined reference of *T. aestivum* plus *Z. tritici*) were created using the Kallisto v0.44.0 [52] Index function, using the default K-mer size of 31. Clean RNAseq reads were mapped to both reference indices and gene abundance was estimated with the Kallisto Quant function, using default parameters. The command used was kallisto quant -i index -o sampleID Sample_1.fq Sample_2.fq, where Sample_1.fq and Sample_2.fq are both paired end reads from the same RNAseq sample. Gene abundance matrices were imported into R using the R package ‘tximport’ [53], using the command and options tximport(files, type = “kallisto”, countsFromAbundance = “scaledTPM”, ignoreAfterBar = TRUE, txIn = TRUE, txOut = TRUE). Options “txIn = TRUE” and “txOut = TRUE” specify that reads were mapped to individual gene variants, rather than genes. All scripts for this analysis are available on GitHub at https://github.com/hbenbow/RNAseq.

### Read count and differential expression analysis

Analysis of read counts was done in R v3.5.2 [54]. Firstly, a matrix of transcript abundance of all gene transcripts x all samples was created, and samples were split by trial. Transcripts were then filtered such that for a given sample (i.e. Stigg, Control, 6 hpi), the transcript was expressed in 2 out of the 3 trials. A Pearson’s correlation analysis was performed using the R function ‘rcorr’. Using the R package “DESeq2” [55], a dds object was created with the design formula ~Treatment + Timepoint + Genotype + Trial + Treatment:Genotype. A variance stabilising transformation was applied to the dds object, and a principle component analysis was performed, using treatment, timepoint, genotype and trial as grouping variables. Differential expression analysis of the *T. aestivum* expression data was done using the R package “DESeq2” [55]. Size factors were estimated for each sample, and the read counts were normalised by the size factor. Dispersion estimates and variance were fitted across all data and differential expression testing was conducted at the gene variant level between treatments per genotype per timepoint.

An independent gene expression study was used for independent biological validation of differentially expressed genes. This study details the Affymetrix *T. aestivum* 61 K Microarray of cv. Stigg and the STB-susceptible cv. Gallant [25]. Probe sequences were retrieved from the Affymetrix website. The IWGSC refseq version 1.1 was formatted into a BLAST nucleotide database using BLAST+ [56], and the Affymetrix probe sequences were BLASTn searched against the IWGSC refseq BLAST database. BLASTn top hits (lowest E-value and highest percentage identity between query and subject sequence) were extracted and each Affymetrix probe was assigned an IWGSC gene ID. Blast2Go was used to identify the biological processes in Stigg and Gallant from the Microarray, and differentially expressed genes from the RNAseq study that were associated with key biological processes were compared to the microarray data for validation of their expression in response to *Z. tritici*.

### Weighted gene co-expression network analysis

Gene network analysis was conducted with the R Package “WGCNA” [26, 57]. The analysis was performed using reads aligned to the combined reference of *T. aestivum* and *Z. tritici* genes across all 48 RNAseq samples. Firstly, a variance stabilizing transformation was applied to the expression data using DESeq2, and the data were transposed into an expression set. The data were split into two expression matrices, one for the Stigg samples and one for the Longbow samples (24 samples per matrix). The two expression matrices were added to a multidata structure and the ‘goodSamplesGenes’ function was used to detect and remove genes and samples with many missing values, or a variance of zero. The filtered expression matrices were then concatenated back into one matrix to choose a soft thresholding power that was appropriate for both subsets of the data. The soft-thresholding power (β) was chosen using the function ‘pickSoftThreshold’, testing candidate powers of 1–10. The soft-thresholding power was chosen as the lowest value for which the scale-free topology index reaches 0.9. Once the appropriate β-value was determined, network construction was conducted on the expression data subsets (Stigg and Longbow separately), using the same value of β for both sets. Due to the large size of the expression matrices, the block-wise network construction and module detection function was used to pre-cluster genes into blocks with a maximum block size of 20,000 genes. The network analysis was then carried out in each block separately using a minimum module size of 30. Module eigengenes (the first principle component of a module) were calculated. Modules were clustered and any modules with a maximum dissimilarity <= 0.25 were merged. To identify modules that are associated with treatment, each sample was given a binary designation of 0 or 1, where control samples were given a 0, and samples treated with *Z. tritici* were given a 1. Module eigengenes were correlated with these trait values and modules with a significant correlation coefficient (*P* < 0.05; 0.5 < =C_c_ < = − 0.5) were retained. Module hubs (i.e. the most connected gene in each module) were identified using the ‘chooseTopHubInEachModule’ function. Module preservation between the Stigg and Longbow networks was calculated using the ‘ModulePreservation’ function in the WGCNA package, firstly using the Longbow expression matrix as the reference set, and the Stigg expression matrix as the test set and secondly using the Stigg data as the reference set and the Longbow data as the test set. For each preserved module, the corresponding modules in the other dataset (i.e. Stigg modules corresponding to a well-preserved Longbow module and vice versa) were defined by identifying the genes within the module, assigning each gene to its corresponding module, and filtering the top 5 modules (i.e. the 5 modules with the most genes in them). These corresponding modules were visualised in the R package ‘igraph’, using the number of genes shared between two modules as the weighting for the edges.

### Gene annotation and ontology analysis

All gene ontology analyses and gene enrichment analyses were done in Blast2GO v5.2.5 [58, 59]. For the gene enrichment analysis, the test set was a list of genes from the condition of interest (i.e. Longbow, up-regulated at 6 h), and the reference set was the complete set of expressed genes detected in the RNAseq data. BLASTn searches were done against the NCBI non-redundant nucleotide database.

### Statistical analysis

All statistical tests were analysed in R v3.5.2 [54]. The distribution of the phenotype data was tested with a Shapiro Wilks test. Transformation of the data to normality was unsuccessful, so the phenotype data was analysed by a Kruskall-Wallis test. A pairwise Wilcox test was used for all pairwise comparisons and to correct *P*-values using the false discovery rate [58]. Chi-square analysis was performed in Microsoft Excel using the chi.test function. All scripts for analysis in this project are available on GitHub at https://github.com/hbenbow/RNAseq.

## Supplementary information

**Additional file 1.**The number of expressed *T. aestivum* reads by subgenome and *Z. tritici* genes in the RNAseq samples.

**Additional file 2.**All differentially expressed wheat genes in cvs. Stigg and Longbow, at 6, 24, 48, and 96 h post inoculation with *Z. tritici.*

**Additional file 3.** Blast and gene ontology annotation of all differentially expressed genes.

**Additional file 4.** Gene expression data for genes within this study that correspond to differentially expressed probes from the published microarray of Brennan et al. [25].

**Additional file 5.** Gene expression data for defence related transcription factors that were up-regulated in the STB-susceptible cultivar Longbow.

**Additional file 6.** Module assignment of genes within the cv. Longbow-specific gene networks.

**Additional file 7.** Module assignment of genes within the cv. Stigg-specific gene networks.

## Data Availability

The raw data (FASTQ files) supporting the conclusions of this manuscript are available in the NCBI SRA database under the bioproject number PRJNA656427. All data analysed and discussed in this manuscript are available in the online supplemental data.

## Abbreviations

ATP: Adenosine triphosphate
BLAST: Basic Local Alignment Search Tool
Bp: Basepair
C_c_: Correlation coefficient
Cv: Cultivar
DEG: Differentially expressed gene
DMI: Sterol 14a-demethylation inhibitors
DPI: Days post inoculation
FDR: False discovery rate
H_2_O_2_: Hydrogen peroxide
HPI: Hours post inoculation
IWGSC: International Wheat Genome Sequencing Consortium
NCBI: National Centre for Biotechnology Information
OR: Oxidation reduction
PAMP: Pathogen associated molecular pattern
PP: Protein phosphorylation
PTI: PAMP-triggered immunity
PTM: Post translational modification
QoI: Quinone-outside inhibitors
RNAseq: Ribonucleic Acid (RNA) sequencing
ROS: Reactive oxygen species
SDHI: Succinate dehydrogenase inhibitors
SDW: Sterile distilled water
SHW: Synthetic hexaploid wheat
STB: Septoria tritici blotch
subsp.: Subspecies
*T. aestivum*: *Triticum aestivum*
TPM: Transcripts per million
URGI: Unité de Recherche Génomique Info
WGCNA: Weighted gene coexpression network analysis
*Z. tritici*: Zymoseptoria tritici
